# Supplementary material for: Marf- and Opa1-Dependent Formation of Mitochondrial Network Structure Is Required for Cell Growth and Subsequent Meiosis in Drosophila Males
Source: Int J Mol Sci. 2025 Oct 14;26(20):9991. doi: 10.3390/ijms26209991 (PMC12562674; doi:10.3390/ijms26209991)
Supplement: Supplementary file 1 [file ijms-26-09991-s001.zip › ijms-3876085-supplementary.pdf]

Supplementary figure legends

Figure S1. Observation of characteristic mitochondrial morphology of spermatocytes at S2b to S6, and during meiosis I, using super-resolution microscopy. (A1-F1) Structured illumination microscopy (SIM) observation of mitochondria immunostained with Complex V alpha-subunit antibody (magenta in A1, red in B1-D1, white in A2-D2) of premeiotic spermatocytes at S2b (A1), S4 (B1), S5 (C1), and S6 (D). Arrows in A2 and D2 indicate elongated mitochondria, while arrowheads in B2 and C2 indicate shortened mitochondria. (A3-D3) Magnified views of the regions enclosed by squares in A2-D2. Blue indicates DNA. (E1, F1) SIM observation of mitochondria (red in E1, F1, white in E2, F2) in primary spermatocytes undergoing prometaphase I (E1) and anaphase I (F1) expressing GFP-tubulin (green). (E3, F3) Magnified views of the regions enclosed by squares in E2 and F2, respectively. Blue: DNA. Arrows in E3 and F3 indicate elongated mitochondria. Bars: 1 μm. (G1, H1) Transmission electron microscopic (TEM) observation of a primary spermatocyte undergoing anaphase I (G1), and telophase I (H1). (G2, H2) Magnified views of areas enclosed by dotted squares in G1 and H1, respectively. Arrows in G1 and H1 point to an example of elongated mitochondria. Bars in G1, H1: 5 μm. Bars in G2, H2: 2 μm.

Figure S2. Time-lapse observation of mitochondria in living spermatocytes, in which the active electron transport membrane potential of mitochondria was maintained, from prophase to telophase of meiosis I.
Time-lapse observation of a spermatocyte undergoing meiosis I. Microtubules in the cells expressing GFP-Tubulin were labelled by GFP fluorescence to visualize microtubules (green). The cell was stained with Mito Tracker to visualize active mitochondria (red). T = 0': time at which GFP fluorescence at cell poles became intense, associated with initiation of active formation of microtubule asters. According to the microtubule structures in meiosis I, the cells were at prometaphase I (t = 20'), metaphase I (t = 30'), late anaphase to early telophase I (t = 40'), and cytokinesis (t = 50'). Mitochondria were partitioned into two daughter cells along microtubules while maintaining the active membrane potential, necessary for ATP synthesis. Bar: 10 μm

Figure S3. The qRT-PCR that quantifies the mRNA levels of *Opa1*, *Marf*, *Drp1*, and *EndoB* in testes harboring ectopic expression of dsRNAs against the relevant mRNAs.
To confirm that the relevant mRNAs were significantly reduced in the knockdown testes,

the qRT-PCR was performed using total RNAs prepared from control male larvae and the knockdown males (A: *Opa1RNAi*^{KK}), (B: *MarfRNAi*^{GD}), (C: *Drp1RNAi*^{JF}), and (D: *EndoBRNAi*^{KK}). The average level in control is presented as 1.0. Error bars indicate the standard error of the mean.

Figure S4. A failure of mitochondrial network structure characteristic of mature spermatocytes at the latest stage, by a knockdown of *Opa1* and *EndoB* but not *Drp1*, was detected by super-resolution microscopy.

(A-C) Observation of mitochondria in mature spermatocytes at S6, immunostained with Complex V alpha-subunit antibody (red in A-C), under a super-resolution microscopy, N-SIM (structured illumination microscopy). Blue indicates DNA. (A) Spermatocytes with knockdown of *Opa1* (*Opa1RNAi*^{KK}), (B) spermatocytes with knockdown of *Drp1* (*Drp1RNAi*^{JF}), and (C) spermatocytes with knockdown of *EndoB* (*EndoBRNAi*^{KK}). Note that the elongated mitochondria are maintained in B, while shortened granule-like mitochondria and slightly shorter mitochondria than those in B are seen in A and C, respectively. Bars in A-C; 5 μm.

Figure S1

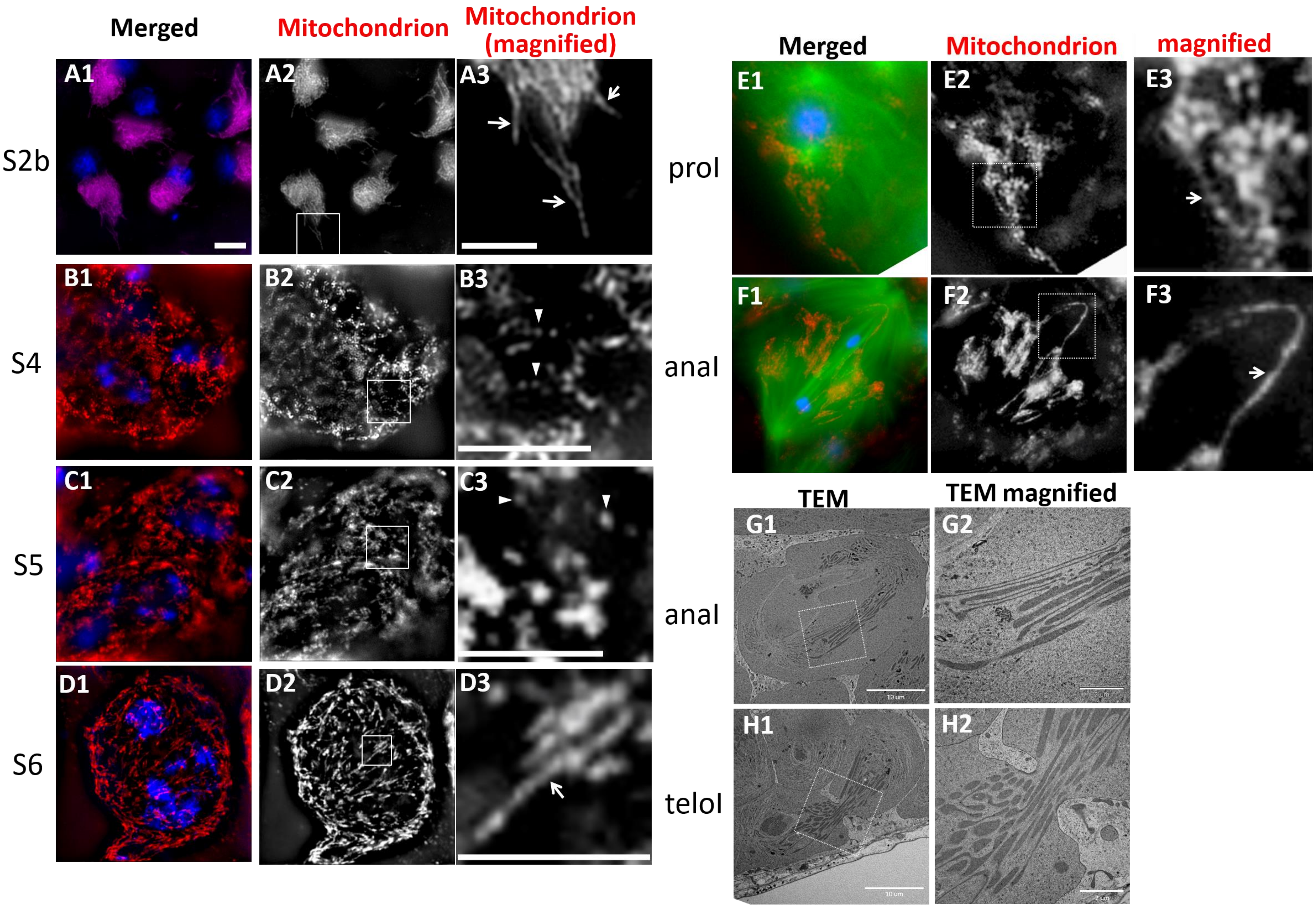
Merged
Mitochondrion
Mitochondrion (magnified)
S2b
S4
S5
S6
A1
A2
A3
B1
B2
B3
C1
C2
C3
D1
D2
D3
Merged
Mitochondrion
magnified
prol
anal
anal
telol
E1
E2
E3
F1
F2
F3
TEM
TEM magnified
G1
G2
H1
H2

Figure S2

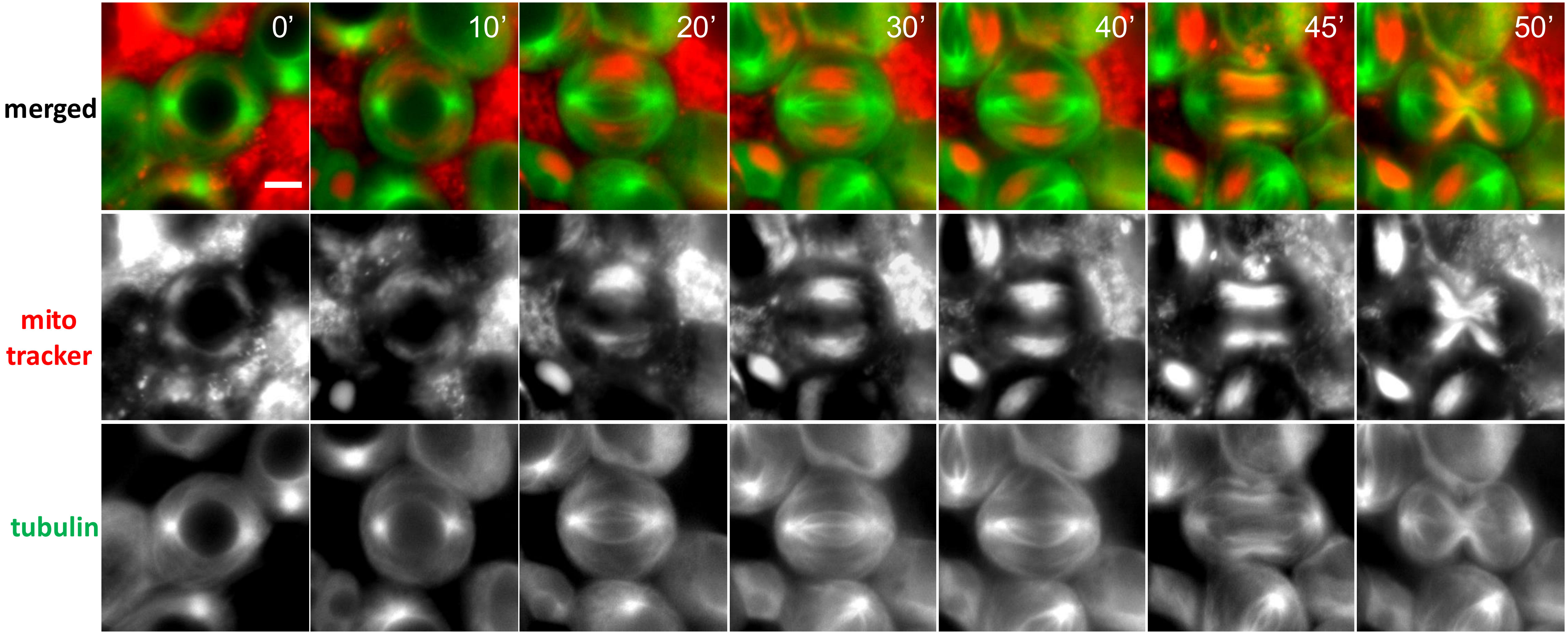

Figure S3

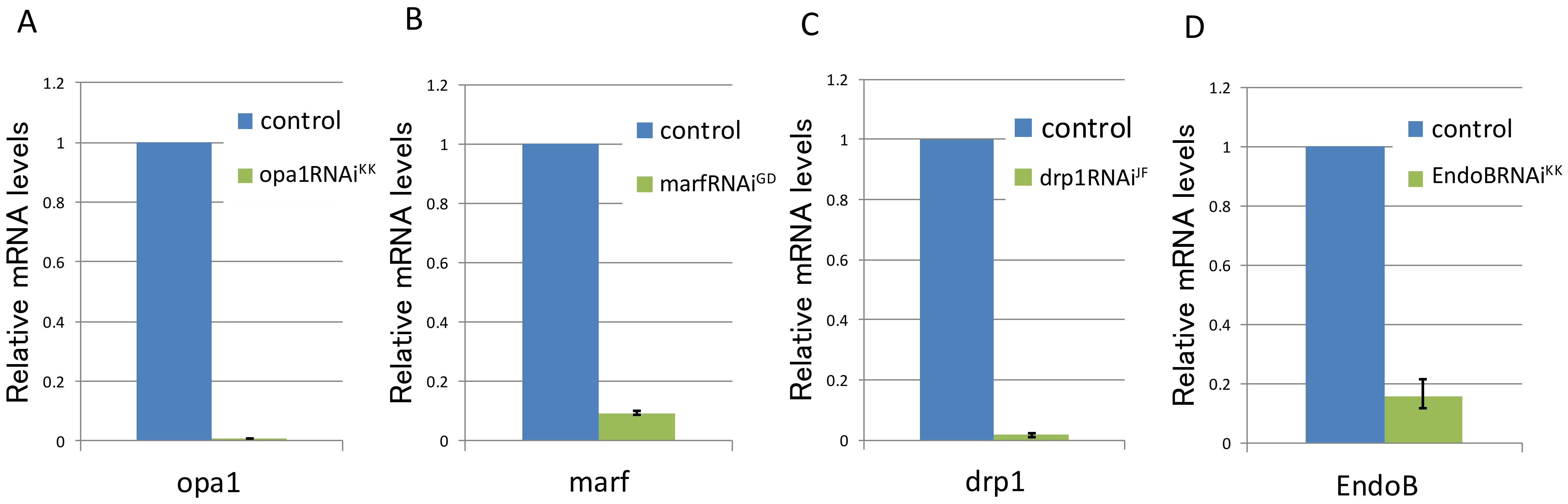

A
B
C
D
Relative mRNA levels
1.2
1
0.8
0.6
0.4
0.2
0
control
opa1RNAi^KK
opa1
marfRNAi^GD
marf
drp1RNAi^JF
drp1
EndoBRNAi^KK
EndoB

Figure S4

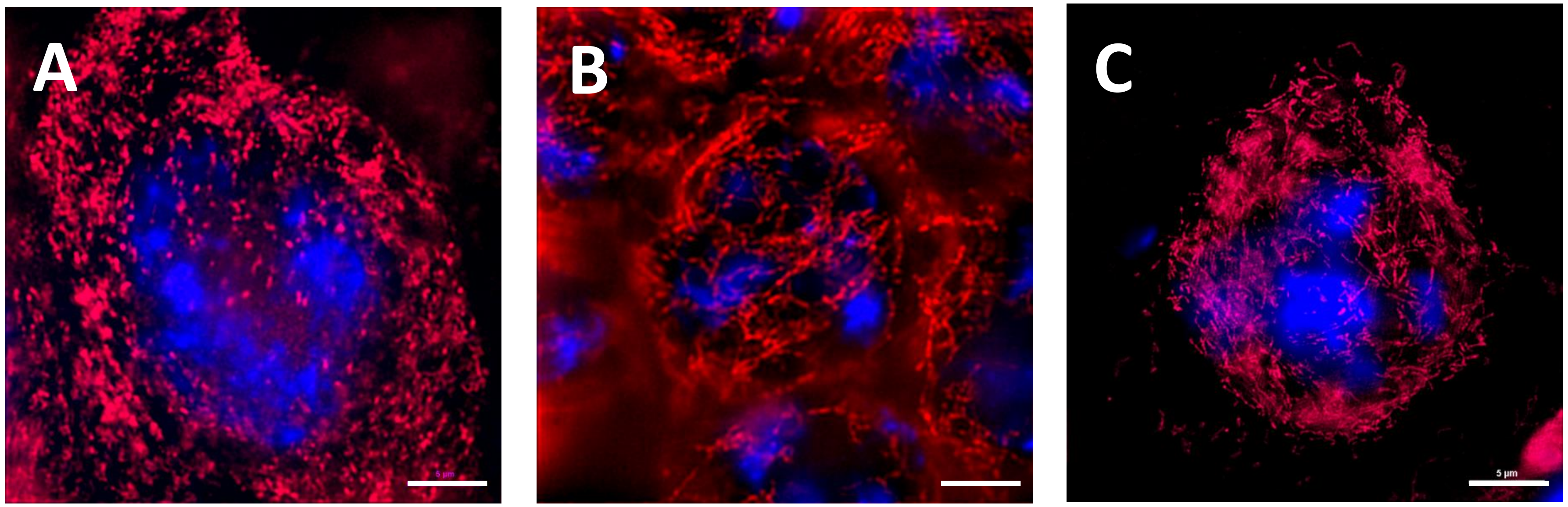

Opa1RNAi *Drp1RNAi* *EndoBRNAi*

Table S1. *Effect of knockdown of the mitochondrial fusion factors and the morphology proteins on the ATP synthesis in testes.*

Genotype	Average ATP amount in the testes (mmol/mg protein) ± *SEM*	Number of male flies used (assays)	Statistical significance*
Control (*bam-Gal4>+)*	65.6 ± 8.4	10 (4)	–
bam>MarfRNAiJF	30.9 ± 0.7	10 (3)	$p < 0.01$
bam>MarfRNAiGD	35.9 ± 0.5	10 (3)	$p < 0.01$
bam>Opa1RNAiHMS	29.2 ± 0.4	10 (3)	$p < 0.01$
bam>Opa1RNAiKK	31.2 ± 0.5	10 (3)	$p < 0.01$
bam>EndoBRNAiKK	6.2 ± 0.3	10 (3)	$p < 0.01$
bam>EndoBRNAiGD	7.0 ± 0.3	10 (3)	$p < 0.01$
bam>Letm1RNAiHMS	10.5 ± 0.2	10 (3)	$p < 0.01$
bam>Letm1RNAiGD	10.4 ± 0.6	10 (3)	$p < 0.01$
bam>blw	7.9 ± 0.3	10 (3)	$p < 0.01$

*Student's t test was used to assess the statistically significant difference between the average lengths of control spermatocytes (bam>+) and cells of each genotype.

Table S2. *Drosophila* stocks used in this study.

Genotype	Abbreviation	Code number	Source	Reference
w^{1118}*;P{GD11094}v40478*	*UAS-MarfRNAi*^{GD}	v40478	VDRC	Poole et al., 2008
y^{1} *v*^{1}*; P{TRiP.JF01650}attP2*	*UAS-MarfRNAi*^{JF}	BL31157	BDSC	Ratnaparkhi, 2013
P{KK106290}VIE-260B	*UAS-Opa1RNAi*^{KK}	v106290	VDRC	Shin et al., 2024
y^{1} *sc* v*^{1} *sev*^{21}*; P{TRiP.HMS00349}attP2*	*UAS-Opa1RNAi*^{HMS}	BL32358	BDSC	Yang et al., 2013
y^{1} *v*^{1}*; P{TRiP.JF02762}attP2*	*UAS-Drp1RNAi*^{JF}	BL27682	BDSC	Sênos Demarco, et al., 2019
w^{1118}*; P{GD2208}v6662/TM3*	*UAS-Letm1RNAi*^{GD}	v6662	VDRC	Schnorrer, et al., 2010
y^{1} *sc* v*^{1} *sev*^{21}*; P{TRiP.HMS01644}attP40*	*UAS-Letm1RNAi*^{HMS}	BL37502	BDSC	Li et al., 2020
w^{1118}*; P{GD14693}v29290*	*UAS-EndoBRNAi*^{GD}	v29293	VDRC	Zhu, et al., 2013
P{KK107885}VIE-260B}	*UAS-EndoBRNAi*^{KK}	v104712	VDRC	FBst0476558
w; P{bam-GAL4::VP16}	*bam-Gal4*	—	E. Mathe	Kitazawa et al., 2012
P{Ubi-tub56D-GFP}	*GFP-tubulin*	—	lab stock	Inoue et al., 2004
M{UAS-CycB.ORF.3xHA}	*UAS-CycB*	#F001154	lab stock	Yamazoe et al., 2023
w; P{UAS-Drp1^{K38A}*}*	*UAS-Drp1*^{DN}	—	J. Chung	Park, et al., 2009
twe^{1} *cn*^{1} *bw*^{1}*/CyO*	*twe*	BL4274	BDSC	White-Cooper et al., 1993
sa^{1} *red*^{1}*/TM3, Sb*^{1}	*sa*	BL1762	BDSC	Lin et al., 1996
